# Enhanced autophagy contributes to excitotoxic lesions in a rat model of preterm brain injury

**DOI:** 10.1038/s41419-018-0916-z

**Published:** 2018-08-28

**Authors:** Céline Descloux, Vanessa Ginet, Coralie Rummel, Anita C. Truttmann, Julien Puyal

**Affiliations:** 1 Department of Fundamental Neurosciences, University of Lausanne, Lausanne, Switzerland; 2 Clinic of Neonatology, Department of Women, Mother and Child, University Hospital Center and University of Lausanne, Lausanne, Switzerland

## Abstract

Cystic periventricular leukomalacia is commonly diagnosed in premature infants, resulting from severe hypoxic-ischemic white matter injury, and also involving some grey matter damage. Very few is known concerning the cell death pathways involved in these types of premature cerebral lesions. Excitotoxicity is a predominant mechanism of hypoxic-ischemic injury in the developing brain. Concomitantly, it has been recently shown that autophagy could be enhanced in excitotoxic conditions switching this physiological intracellular degradation system to a deleterious process. We here investigated the role of autophagy in a validated rodent model of preterm excitotoxic brain damage mimicking in some aspects cystic periventricular leukomalacia. An excitotoxic lesion affecting periventricular white and grey matter was induced by injecting ibotenate, a glutamate analogue, in the subcortical white matter (subcingulum area) of five-day old rat pups. Ibotenate enhanced autophagy in rat brain dying neurons at 24 h as shown by increased presence of autophagosomes (increased LC3-II and LC3-positive dots) and enhanced autophagic degradation (SQSTM1 reduction and increased number and size of lysosomes (LAMP1- and CATHEPSIN B-positive vesicles)). Co-injection of the pharmacological autophagy inhibitor 3-methyladenine prevented not only autophagy induction but also CASPASE-3 activation and calpain-dependent cleavage of SPECTRIN 24 h after the insult, thus providing a strong reduction of the long term brain injury (16 days after ibotenate injection) including lateral ventricle dilatation, decreases in cerebral tissue volume and in subcortical white matter thickness. The autophagy-dependent neuroprotective effect of 3-methyladenine was confirmed in primary cortical neuronal cultures using not only pharmacological but also genetic autophagy inhibition of the ibotenate-induced autophagy. Strategies inhibiting autophagy could then represent a promising neuroprotective approach in the context of severe preterm brain injuries.

## Introduction

The important progress done in neonatal care constantly increases the survival rates of premature infants. Conversely, the proportion of neurological disabilities developed by survivors is hardly reduced especially for those with severe impairment. One of them is the diplegic cerebral palsy, called also spastic diplegia of Little^1^, affecting still between 3–7% of very low birth weight (VLBW) infants^2,3^. The strongest predictor of this form of cerebral palsy in VLBW infants is cystic periventricular leukomalacia (cPVL)^4^, a form of preterm white matter (WM) injury adjacent to the lateral ventricles, which occurs either from a hypoxic-ischemic (HI) event around birth or after infectious events such as septic shock, necrotizing enterocolitis or even reported after viral infections^5,6^. Improving the outcomes for these severely affected babies remains a challenging health issue.

Beside inflammation and reactive oxygen species formation, excitotoxicity seems to be crucial in the pathophysiology of many preterm brain injuries such as PVL^3,7,8^. Excitotoxicity consists in an excessive or prolonged activation of excitatory amino acid receptors (especially those of glutamate) due to a failure of sufficient reuptake and/or excessive release at the synaptic level. It induces a massive increase in intracellular calcium concentration and thus activates numerous intracellular cascades potentially leading to neuronal cell death^9,10^. Glutamate homeostasis is highly important for human brain development (proliferation, migration, differentiation, survival processes and synapses refinement)^11^. However it also confers to immature brain a vulnerability to excitotoxic injuries since a higher level of ionotropic glutamate receptors are expressed in developing brain compared to that of adult^8,12–15^. These receptors are in addition more readily activated. Excitotoxic lesions can occur following a panel of deleterious events (that can be related) such as infection/inflammation, hypoxia and/or ischemia. Excitotoxicity is then a common pathological mechanism of various perinatal brain injuries. In neurons the peak of expression of NMDA receptors appears to occur at term in which grey matter (GM) damage is predominant than in preterm^13^. In human WM, this peak occurs in preterm brain glial cells, especially in pre-oligodendrocytes O4^+^, and contributes to the high sensitivity of preterm WM. PVL was mainly thought to be associated to WM injury but it is clearly shown now that GM damage is also often involved in a “neuronal-axonal disease”^16,17^.

Experimental research has revealed the complexity of the pathophysiology of excitotoxic death showing multiple and interrelating cell death mechanisms reflected by mixed features of neuronal death including not only the well-known “apoptotic-necrotic continuum” with features of apoptosis and necrosis in the same dying neurons^18^ but also characteristics of enhanced macroautophagy^10,19–21^. Autophagy is a physiological cellular mechanism of degradation and recycling of dysfunctioning long lived proteins and organelles^22^. Its main form (macroautophagy, hereafter called autophagy), consists in the formation of a multimembrane intermediate compartment, named autophagosome, that engulfs part of the cytosol containing proteins and organelles to be degraded. The autophagosome then fuses with a lysosome, forming an autolysosome, to degrade its content through lysosomal hydrolases activity^22^. Autophagy is thus essential for cellular homeostasis and could be used as a survival response to different stresses such as nutrients deprivation, accumulation of toxic proteins or pathogen invasion^23^. However, dysregulated increase in autophagic process has been also implicated in cell death as an independent mechanism (termed “autophagic cell death”) or more frequently as a mediator of other types of cell death, mainly apoptosis, and then designed as “autophagy-mediated cell death”^10,24–28^. Abnormal high level of autophagosomes and autolysosomes with increased lysosomal enzyme activity were often observed in dying neurons in models of excitotoxicity including perinatal cerebral HI^19,20,29–31^. Interestingly, we also recently demonstrated excessive autophagic features in postmortem brains of human term newborns presenting severe hypoxic-ischemic encephalopathy (HIE)^29,31^. Although controversies remain concerning the role of autophagic activation^24,32–35^, most of the studies using autophagy inhibition, either through pharmacological inhibitors such as 3-methyladenine (3-MA)^30,36–40^ or through specific and genetic inhibition of autophagy-related genes (*atg*)^20,29,31,41^, have revealed a pro-death role of autophagy in perinatal and adult cerebral HI models.

The present study aims to determine the role of autophagy in excitotoxic lesions of the premature brain using a widely recognized rodent model that mimics some features of cPVL^42^. Autophagy flux and the neuroprotective effect of autophagy inhibition either pharmacologically with 3-MA or genetically in neuronal cultures was investigated in the context of an excitotoxic insult induced by an injection of the glutamate analogue ibotenate. An involvement of autophagy in excitotoxic preterm brain damage would reveal a new cellular death pathway and open the way for new neuroprotective strategies in severe preterm brain injuries.

## Results

### Ibotenate injection in subcortical WM of rat pups induces brain injury

**Fig. 1:**
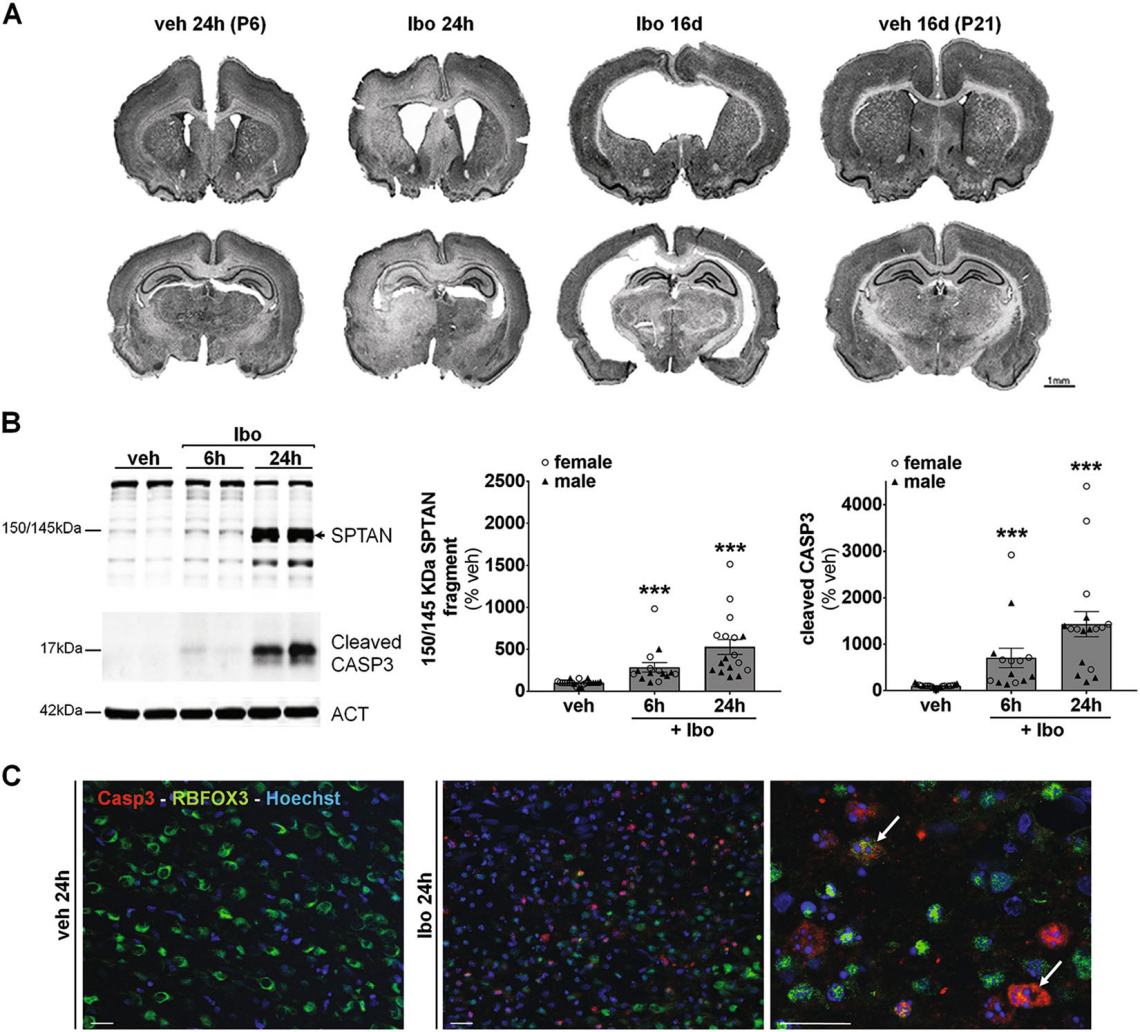
Ibotenate injection in subcortical white matter of rat pups induces brain injury. **a** Representative cresyl violet-stained coronal sections illustrating the brain damage 24 h (at 6 postnatal days (P6)) and 16 days (P21) after ibotenate (Ibo) injection. Scale bar: 1 mm. **b** Ibo injection activates both 150–145 kDa calpain-dependent cleavage of spectrin/fodrin (SPTAN) (veh: 100 ± 6.3%, 6 h Ibo: 284.4 ± 56.6%, 24 h Ibo: 528.1 ± 88.8%) and those of caspase-3 (CASP3) (veh: 100 ± 7.13%, 6 h Ibo: 702.6 ± 210%, 24 h Ibo: 1430.9 ± 271.1%) as shown by representative immunoblots and the corresponding quantifications. Mean CASP3 value is 870.7 ± 204.1% for males and 1929 ± 423.5% for females at 24 h. Mean SPTAN value is 312.1 ± 55.9% for males and 720.1 ± 132.5% for females at 24 h. (veh: *n* = 11 females, 10 males; 6 h: *n* = 7 females, 7 males; 24 h: *n* = 9 females, 8 males). Males are represented by black triangles and females by white circles. **c** CASP3 is activated in neurons as revealed by double immunolabeling against cleaved CASP3 (in red) and the neuronal marker RNA Binding Protein, Fox-1 Homolog 3 (RBFOX3)/NeuN (in green) 24 h after Ibo injection in neurons. Scale bar: 20 µm. Values are mean ± SEM, *p < 0.05; **p < 0.01; ***p < 0.001

As a model mimicking some aspects of cPVL, we selected a widely recognized model of preterm excitotoxic brain injury consisting in applying an intracerebral injection of ibotenate in rat pups^42^. The injection of ibotenate (10 µg) in the subcortical WM at the level of the right cingulum of 5-days-old rat pups causes a severe brain damage of the WM and GM resulting in ventricular enlargement as illustrated by cresyl-violet-stained coronal sections in Fig. 1a 24 h and 16 days after the insult. Immunoblot analysis of two death markers, the calpain-dependent 150–145 kDa spectrin/fodrin (SPTAN) fragment and cleaved CASPASE-3 (CASP3), indicated an activation of both calcium-dependent necrotic cell death and caspase-dependent apoptosis (Fig. 1b) at 6 and 24 h after the insult (~5 and 14 fold increases respectively at 24 h). Although the profile of CASP3 activation was similar between females and males, we observed 24 h after the insult a greater activation (~2 times more) in females. Double immunolabeling showed that CASP3 was activated in neurons (RBFOX3-positive cells) 24 h after ibotenate injection (Fig. 1c).

### Ibotenate injection in subcortical WM of rat pups enhances autophagic flux in cortical neurons

**Fig. 2:**
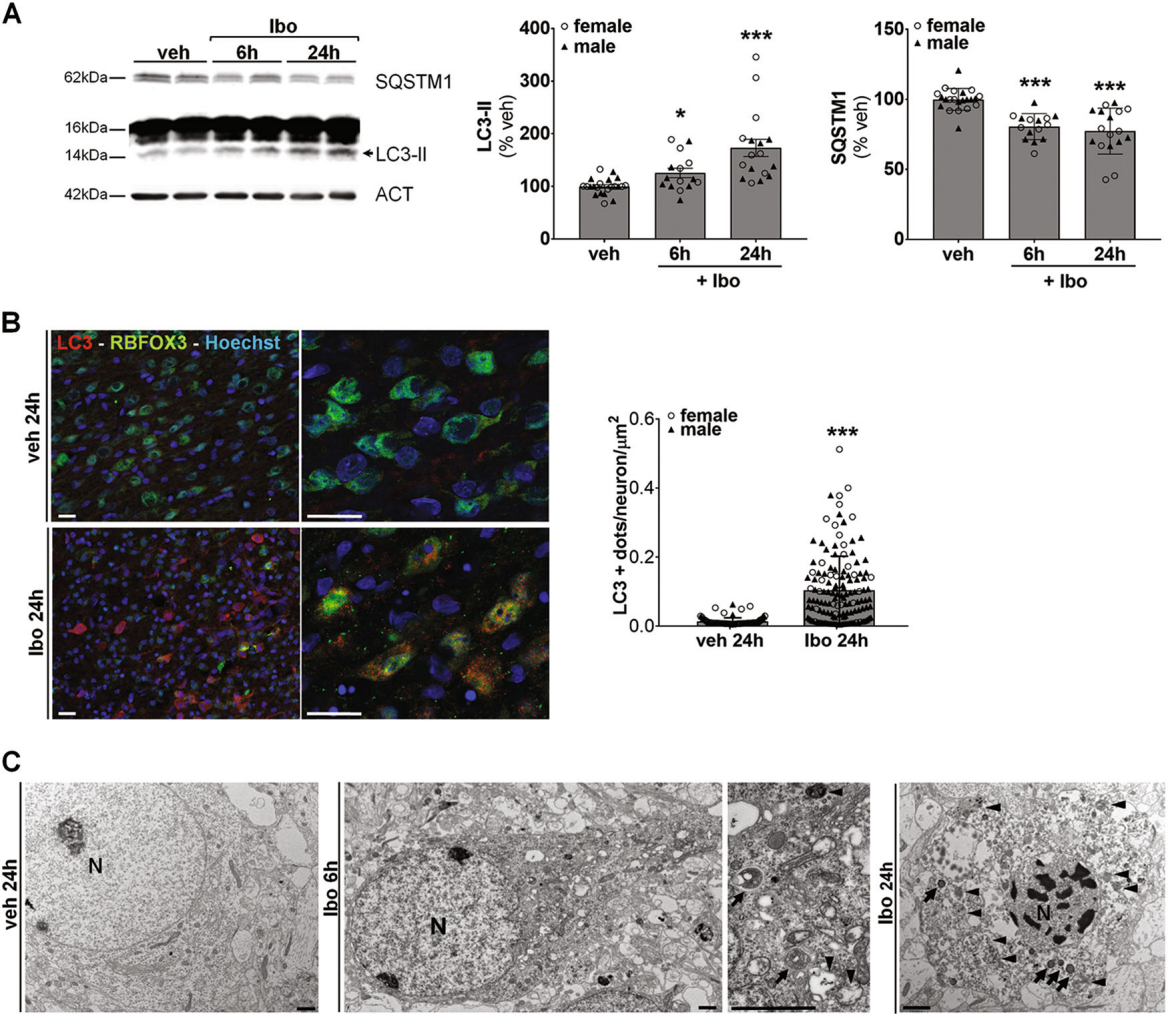
Ibotenate injection in subcortical white matter of rat pups enhances autophagy in neurons. **a** Representative immunoblots and the corresponding quantifications demonstrate an increase in LC3-II (veh: 100 ± 3.3%, 6 h Ibo: 125.6 ± 8.9%, 24 h Ibo: 173.2 ± 16.4%) and a decrease in SQSTM1 (veh: 100 ± 1.7%, 6 h Ibo: 80.5 ± 2.4%, 24 h Ibo: 77.3 ± 4%) level of expression from 6 h after ibotenate (Ibo) injection. Mean LC3-II value is 146.9 ± 12% for males and 196.6 ± 27.5% for females at 24 h. Mean SQSTM1 value is 81 ± 4.4% for males and 74 ± 6.5% for females at 24 h. (veh: *n* = 11 females, 10 males; 6 h: *n* = 7 females, 7 males; 24 h: *n* = 9 females, 8 males). **b** Quantification of LC3-positive dots (in red) per neuron ((RNA Binding Protein, Fox-1 Homolog 3) RBFOX3/NeuN (in green)) per µm^2^ on confocal images shows an increase in the number of autophagosomes in the cortex 24 h after Ibo injection (veh: 0.0131 ± 0.001, Ibo: 0.1039 ± 0.0083). Mean LC3-positive dots per neuron per µm2 value is 0.1366 ± 0.0137% for males and 0.0711 ± 0.0079% for females treated with Ibo. Males are represented by black triangles and females by white circles. Values are mean ± SEM, *p < 0.05; **p < 0.01; ***p < 0.001. Scale bars: 20 µm. **c** Ultrastructure analysis of damaged cortex reveals the presence of numerous autophagosomes (multimembrane vacuoles, arrow heads) and autolysosomes (electron dense structures, arrows) in the cytosol at 6 and 24 h. Nuclear chromatin condensation and swollen organelles are observed at later stage (24 h). Nucleus (N). Scale bars: 1 µm

To study the effect of intracerebral injection of ibotenate on autophagy, the expression levels of LC3-II (the autophagosomal membrane bound form of LC3) and Sequestosome-1 (SQSTM1, selectively degraded by autophagy) in ipsilateral cortical extracts was first analyzed at 6 and 24 h after the ibotenate injection (Fig. 2a). LC3-II was significantly enhanced by ibotenate compared to vehicle treated-rats (increase of 72% at 24 h) whereas SQSTM1 was decreased (reduction of 23% at 24 h) suggesting an increase in the autophagy flux in females as well as in males. A quantification of the number of LC3-positive dots per neuron (RBFOX3-positive cells) confirmed a significant increase of 7.9 fold in the number of autophagosomes in cortical neurons 24 h after the insult (Fig. 2b). Moreover, strong morphological features of enhanced autophagy could be observed at the ultrastructural level (Fig. 2c). At earlier stage (6 h after ibotenate injection), electron microscopy revealed numerous multimembrane structures containing undigested cellular contents (autophagosomes) and electron dense vacuoles containing cytoplasmic materials at different stages of degradation (autolysomes) whereas later on (at 24 h) hybrid phenotypes of cell death are observed in dying neurons including apoptosis-like morphological features (chromatin condensation), enhanced autophagy (numerous autophagosomes and autolysosomes) and swollen organelles.

**Fig. 3:**
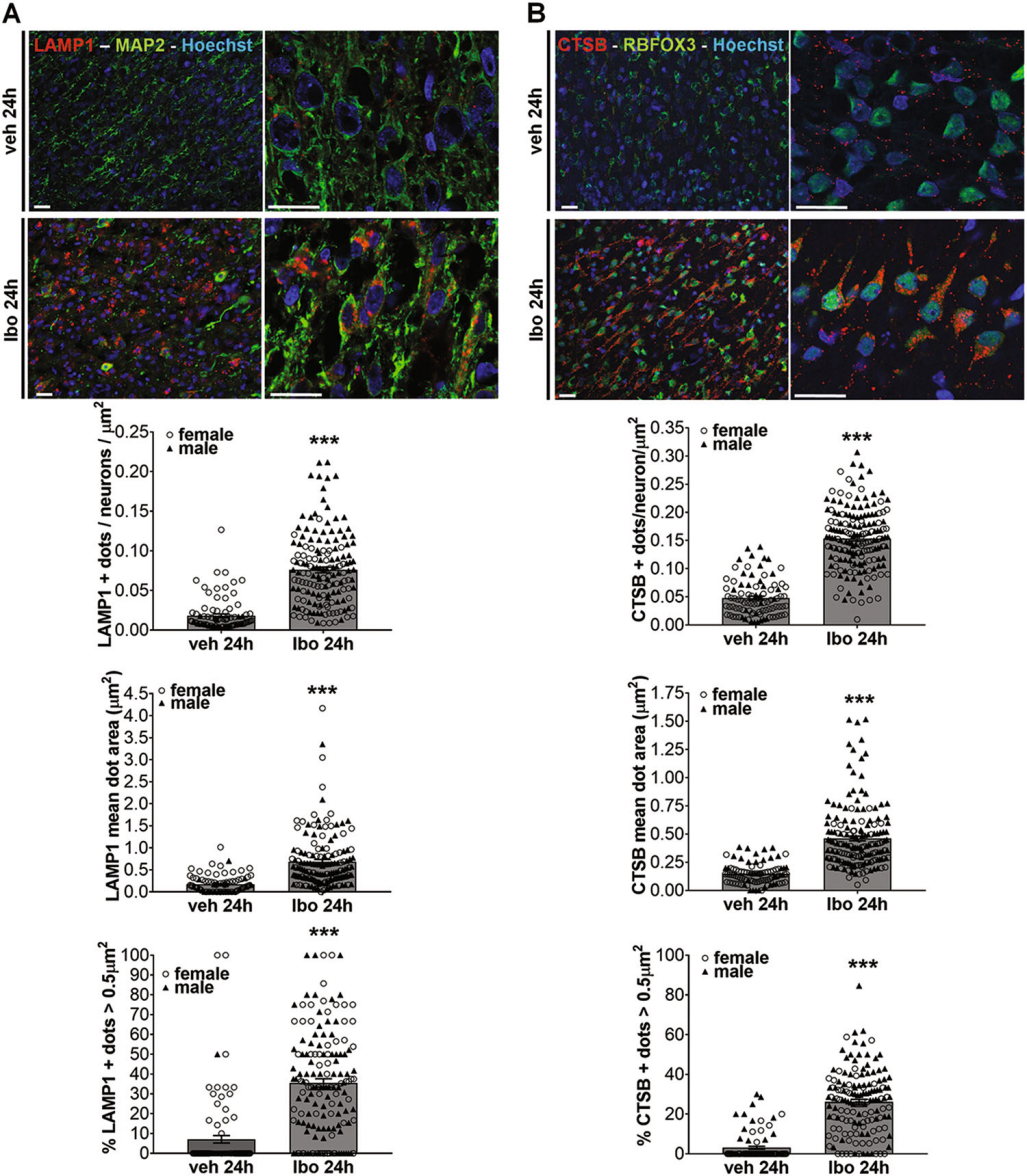
Ibotenate injection in subcortical white matter of rat pups increases autolysosomes formation in neurons. **a** Quantification of LAMP1-positive dots (in red) per neuron (MAP2 (in green)) per µm^2^ on confocal images shows an increase in the number of LAMP1-positive lysosomes in the cortex 24 h after Ibo injection (number of dots: veh: 0.018 ± 0.002, Ibo: 0.075 ± 0.003/neurons/µm^2^). Mean value is 0.089 ± 0.005% for males and 0.057 ± 0.004% for females treated with Ibo. On average, the measure of their size reveals the presence of larger LAMP1-positive dots following Ibo injection (mean dot area: veh: 0.171 ± 0.002, Ibo: 0.685 ± 0.049 µm^2^) resulting from more dots larger than 0.5 µm^2^ (veh: 7.08 ± 1.90%, Ibo: 35.56 ± 2.08%). Mean value is 0.571 ± 0.051 µm^2^ / 38.3 ± 3.4% for males and 0.849 ± 0.091 µm^2^/38.3 ± 3.4% for females treated with Ibo. **b** Quantification of CATHEPSIN B (CTSB)-positive dots (in red) per neuron ((RNA Binding Protein, Fox-1 Homolog 3) RBFOX3/NeuN (in green)) confirms an increase not only in the number (number of dots: veh_M+F_: 0.047 ± 0.003, Ibo_M+F_: 0.153 ± 0.004, Ibo_M_: 0.153 ± 0.005, Ibo_F_: 0.153 ± 0.007/neurons/µm^2^) but also in the size of lysosome (mean dot area: veh_M+F_: 0.146 ± 0.009, Ibo_M+F_: 0.460 ± 0.020 µm^2^, Ibo_M_: 0.490 ± 0.025 µm^2^, Ibo_F_: 0.343 ± 0.021 µm^2^; dots > 0.5 µm^2^: veh_M+F_: 3.033 ± 0.723%, Ibo_M+F_: 26.10 ± 1.274%, Ibo_M_: 26.8 ± 1.3%, Ibo_F_: 18.6 ± 2.1%). Scale bars: 20 µm. Values are mean ± SEM, *p < 0.05; **p < 0.01; ***p < 0.001. (veh: *n* = 63 females, 30 males; Ibo: *n* = 35 females, 140 males). Males are represented by black triangles and females by white circles

Finally, in addition to a higher number of autophagosomes, autolysosomes were also more numerous in neurons of ibotenate-treated rat pups than in vehicle-treated animals. Immunohistochemistry and quantification of the number and the size of vesicles labeled with antibodies against either a lysosomal membrane bound protein, lysosomal-associated membrane protein 1 (LAMP1) (Fig. 3a), or a lysosomal enzyme (CATHEPSIN B) (Fig. 3b) demonstrated an increase not only in thenumber but also in the size of either LAMP1 (~4 fold) or CATHEPSIN B (~3 fold) positive-dots, and especially those larger than 0.5 µm^2^ (increase of 29 and 23%, respectively) that are presumably autolysosomes. These results showed that ibotenate resulted in enhancement of neuronal autophagy in the damaged cortex of rat pups.

### Ibotenate induced-autophagy and -cell death are reduced by pharmacological inhibition of autophagy

**Fig. 4:**
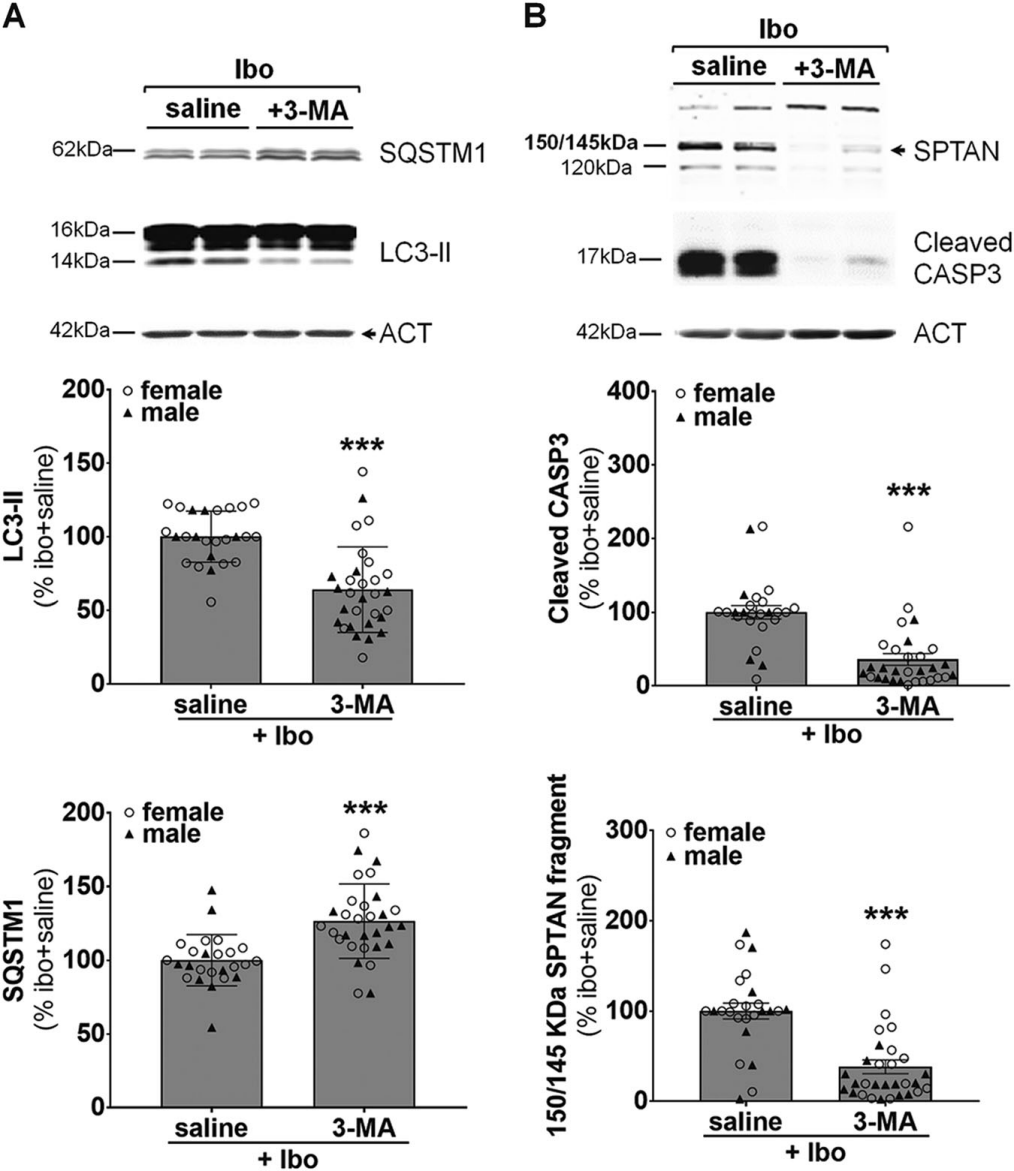
Ibotenate induced-autophagy and cell death is reduced by pharmacological inhibition of autophagy. **a** Intracerebroventricular injection of the autophagy inhibitor 3-methyladenine (3-MA) just before ibotenate (Ibo) treatment decreases LC3-II (Ibo + 3-MA: 64.1 ± 5.3%) and increases SQSTM1 (Ibo + 3-MA: 126.5 ± 4.6%) levels 24 h after Ibo injection compared to rat pups treated with saline (Ibo + saline: LC3, 100 ± 3.5%; SQSTM1, 100 ± 3.5%) as shown by representative immunoblots of cortical extracts and the corresponding quantifications. Mean LC3-II value is 55.6 ± 6.7% for males and 71.5 ± 7.7% for females at 24 h. Mean SQSTM1 value is 127.4 ± 7.2% for males and 127.5 ± 7.07% for females at 24 h. **b** 3-MA prevents both 150–145 kDa calpain-dependent cleavage of spectrin/fodrin (SPTAN) (Ibo + saline: 100 ± 8.8%, Ibo + 3-MA: 51.5 ± 12.9%) and those of caspase-3 (CASP3) (Ibo + saline: 100 ± 9.1%, Ibo + 3-MA: 55.8 ± 14.4%). Mean SPTAN value is 21.7 ± 4.3% for males and 52.7 ± 12.9% for females at 24 h. Mean CASP3 value is 25.8 ± 6.1% for males and 44.8 ± 13.7% for females at 24 h. Values are mean ± SEM, *p < 0.05; **p < 0.01; ***p < 0.001. (Ibo + saline: *n* = 15 females, 10 males; Ibo + 3-MA: *n* = 16 females, 14 males). Males are represented by black triangles and females by white circles

We then evaluated the role of this enhanced neuronal autophagy on brain damage. Since long term autophagy inhibition could be potentially deleterious particularly for neurons^43^, we decided to manage the transient inhibition of autophagy with the widely used pharmacological inhibitor of autophagy 3-MA. 3-MA was administered in the ipsilateral ventricle just before ibotenate injection as previously done in ischemic rat pups^30^. As shown by immunoblots, ibotenate-induced increase in LC3-II expression levels at 24 h was reduced by 36% in 3-MA-treated rats compared to saline-injected rats (Fig. 4a). Moreover, SQSTM1 expression levels were also significantly increased by 26% with 3-MA treatment confirming an efficient inhibition of autophagy by 3-MA (Fig. 4a). We then evaluated the effect of autophagy inhibition on cell death markers at 24 h. The expression levels of both the 150–145 kDa SPTAN fragment and cleaved CASP3 were significantly decreased by 64 and 62% respectively with 3-MA treatment (Fig. 4b) demonstrating that autophagy inhibition by 3-MA could be neuroprotective 24 h after the induction of the excitotoxic brain lesion. Moreover 3-MA treatment was as efficient in females as well as in males.

### Long term ibotenate-induced brain damage is strongly reduced by pharmacological inhibition of autophagy

**Fig. 5:**
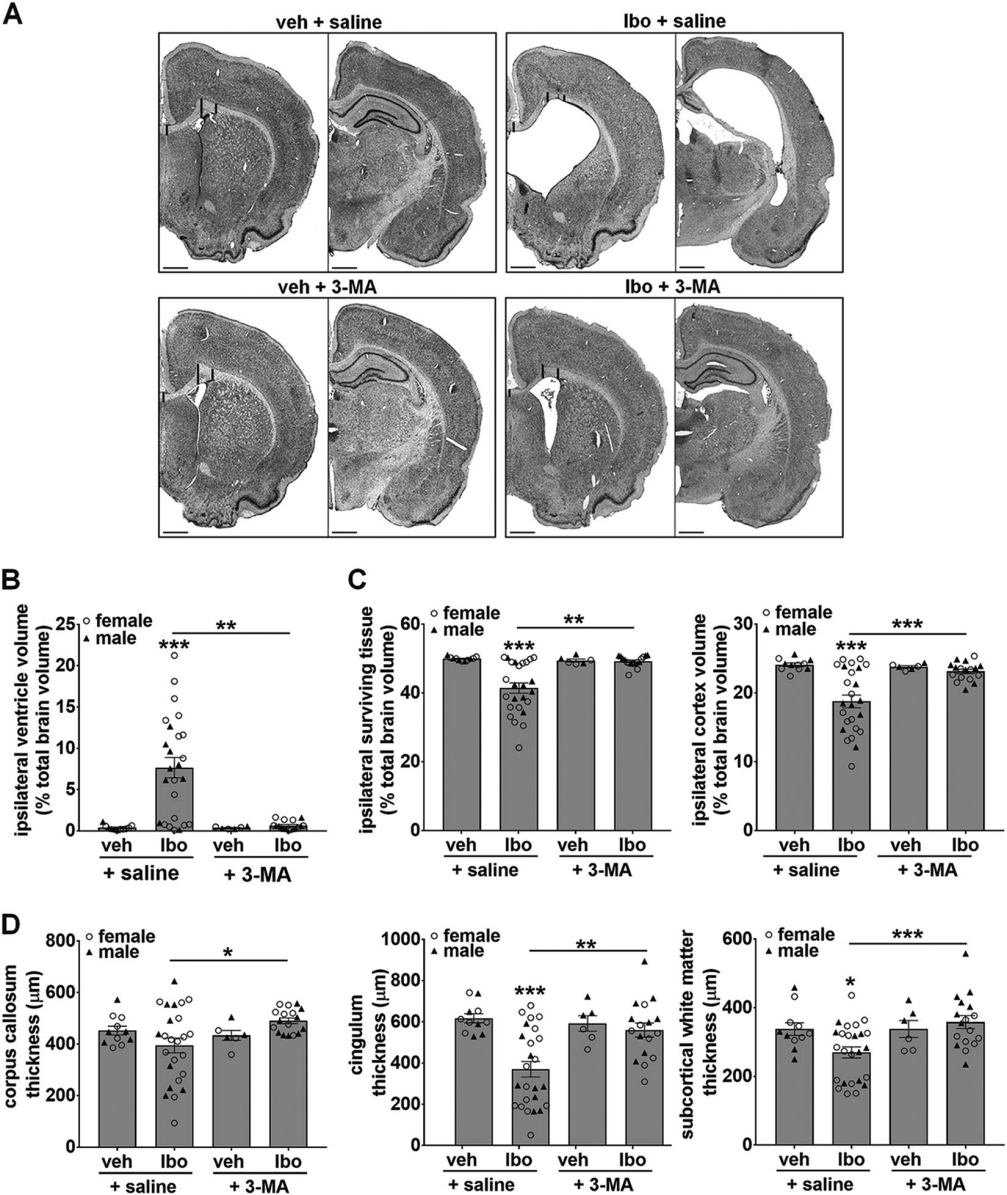
Long term ibotenate-induced brain damage is strongly reduced by pharmacological inhibition of autophagy. **a** Representative cresyl violet-stained coronal sections of brain 16 days (P21) after injury illustrating the protective effect of the autophagy inhibitor 3-methyladenine (3-MA) injected in the ipsilateral ventricle just before ibotenate (Ibo). Scale bar: 1 mm. **b** 3-MA treatment reduces Ibo-induced ventricle dilatation (veh + saline: 0.386 ± 0.086%, Ibo + saline: 7.655 ± 1.233%, Ibo + 3-MA: 0.646 ± 0.112%, veh + 3MA: 0.366 ± 0.056%). Mean values for Ibo + saline treatment are 6.88 ± 1.38% for males and 8.10 ± 1.79% for females whereas those for Ibo + 3-MA are 0.53 ± 0.16% for males and 0.78 ± 0.21% for females. **c** Quantification of ipsilateral surviving tissue volume (veh + saline: 49.94 ± 0.167%, Ibo + saline: 41.41 ± 1.486%, Ibo + 3-MA: 49.22 ± 0.379%, veh + 3MA: 49.43 ± 0.428%) relatively to total brain volume and more specifically those of the ipsilateral cortex (veh + saline: 24.09 ± 0.258%, Ibo + saline: 18.76 ± 0.926%, Ibo + 3-MA: 23.13 ± 0.329%, veh + 3MA: 23.78 ± 0.170%) shows that Ibo-induced decrease in brain tissue volume is prevented by 3-MA. Mean surviving tissue volumes for Ibo + saline treatment are 42.11 ± 1.68% for males and 41.02 ± 2.16% for females whereas those for Ibo + 3-MA are 49.92 ± 0.34% for males and 48.45 ± 0.59% for females. **d**. 3-MA attenuates the Ibo-induced decrease in the thickness of white matter located at three different levels: corpus callosum (veh + saline: 452.61 ± 17.16 µm, Ibo + saline: 394.72 ± 28.76 µm, Ibo + 3-MA: 490.73 ± 11.08 µm, veh + 3MA: 433.67 ± 19.19 µm), cingulum region (veh + saline: 617.54 ± 21.96 µm, Ibo + saline: 369.76 ± 37.63 µm, Ibo + 3-MA: 560.37 ± 35.46 µm, veh + 3MA: 592.40 ± 37.53 µm) and at the beginning of the external capsule (veh + saline: 337.89 ± 18.44 µm, Ibo + saline: 269.80 ± 16.23 µm, Ibo + 3-MA: 358.28 ± 19.20 µm, veh + 3MA: 338.05 ± 24.81 µm). 3-MA tends to have a similar protective effect on white matter thickness in males and females. Values are mean ± SEM, *p < 0.05; **p < 0.01; ***p < 0.001. (veh + saline: *n* = 6 females, 5 males, Ibo + saline: *n* = 16 females, 9 males, Ibo + 3-MA: *n* = 8 females, 9 males, veh + 3MA: *n* = 3 females, 3 males). Males are represented by black triangles and females by white circles

We then evaluated the long term benefit of 3-MA-mediated autophagy inhibition on brain injury at P21 (16 days after ibotenate injection). As shown in Figs. 5a, 3-MA treated-brains were less sensitive to ibotenate-induced damage. The ventricle enlargement of ~20 fold produced by ibotenate injection was strongly prevented by 3-MA treatment (reduced to only 1.7 fold) (Fig. 5b). Moreover, ibotenate-induced tissue loss of 8.54% (total ipsilateral WM and GM), with a decrease of 5.33% specifically for cortex (total ipsilateral intact cortex), was also, on average, significantly prevented in 3-MA-treated rats compared to saline-treated rats (Fig. 5c).

To evaluate the effect of ibotenate on WM, the thickness of the subcortical WM was measured at three different levels around the ibotenate injection site. Reduced thickness could reflect an indirect effect of the GM lesion with secondary axonal degeneration and/or a direct excitotoxic effect on immature oligodendrocytes. A significant decrease in the thickness of subcortical WM of 13% for corpus callosum, 40% for the WM located at the level of cingulum and 20% for external capsule was detected in ibotenate-treated brains (Fig. 5d). These WM alterations were not observed when 3-MA was co-injected with ibotenate (Fig. 5d).

Taken together, these data showed a long term beneficial effect of pharmacological autophagy inhibition against ibotenate-induced brain damage that was moreover observed both in males and females.

### An excitotoxic dose of ibotenate enhances autophagic flux in primary cortical neuronal cultures

**Fig. 6:**
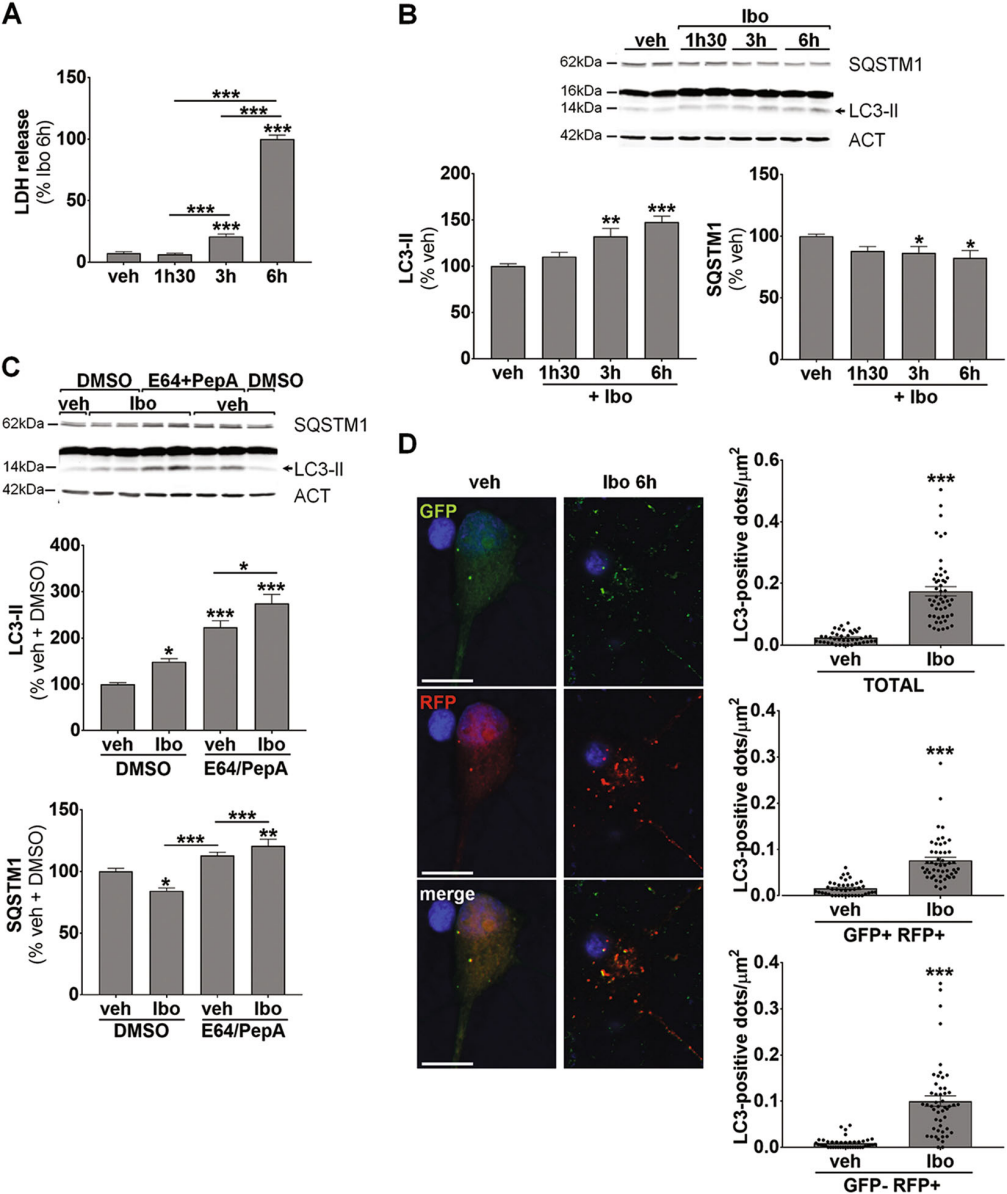
Excitotoxic dose of ibotenate enhances autophagic flux in primary cortical neuronal cultures. **a** Ibotenate (Ibo, 50 µM) is neurotoxic as shown by lactate deshydrogenase (LDH) release in the culture medium of cultured neurons (veh: 7.4 ± 1.1%, 1h30: 6.4 ± 0.9%, 3 h: 21 ± 1.9%, 6 h: 100 ± 3.2%). **b** Representative immunoblots and the corresponding quantifications demonstrate an increase in LC3-II (veh: 100 ± 2.7%, 1h30: 110.4 ± 4.6%, 3 h: 132.3 ± 8.5%, 6 h: 147.6 ± 6.6%) and a decrease in SQSTM1 (veh: 100 ± 1.5%, 1h30: 89.7 ± 3.3%, 3 h: 88.1 ± 4.4%, 6 h: 84.2 ± 4.4%) level of expression following Ibo treatment. **c** Addition of pepstatin A (PepA) and E64 prevents lysosomal degradation as shown by increases in the level of LC3-II (veh Pep/E64: 223.2 ± 14.3%) and SQSTM1 (veh Pep/E64: 113 ± 2.8%) relative to neurons treated with DMSO (veh DMSO; LC3-II: 100 ± 3.3%, SQSTM1: 100 ± 2.6%). When added 4 h before Ibo (3 h), PepA/E64 treatment results in a greater increase in LC3-II (Ibo E64/PepA: 274.1 ± 19.9%) than treatment with E64/PepA or Ibo alone (Ibo DMSO: 148.7 ± 6.7%). E64/PepA pretreatment inhibits the Ibo-induced degradation of SQSTM1 (Ibo DMSO: 84.4 ± 2.2%; Ibo E64/PepA: 120.8 ± 5.5%). **d** Representative confocal images of cultured neurons transfected with the tandem mRFP-GFP-LC3–expressing plasmid 6 h after Ibo addition. Quantification of the number of LC3-positive dots per neuron per µm^2^ demonstrates an enhanced functional autophagic flux (Total = GFP + RFP+ and GFP−RFP+ : veh: 0.024 ± 0.003, Ibo: 0.174 ± 0.016; GFP + RFP + (early autophagosomes): veh: 0.015 ± 0.002, Ibo: 0.076 ± 0.007; GFP−RFP + (autolysosomes) veh: 0.008 ± 0.001, Ibo: 0.100 ± 0.011). Scale bar: 10 µm.Values are mean ± SEM, *p < 0.05; **p < 0.01; ***p < 0.001

In order to confirm that ibotenate could enhance autophagy and that specific autophagy inhibition could be neuroprotective against ibotenate-mediated excitotoxicity, we then decided to evaluate the effect of an excitotoxic dose of ibotenate on autophagy in primary cortical neuronal cultures. In general, 50 µM of ibotenate promoted neuronal death as suggested by a 3 and 14 fold increase in lactate deshydrogenase (LDH) release in the culture medium 3 and 6 h, respectively after ibotenate treatment (Fig. 6a). This LDH release was abolished by MK801 or EGTA pretreatment (data not shown) confirming excitotoxic mechanisms (NMDA receptors overactivation and calcium overload).

As shown in Fig. 6b, activation of autophagy occurred along with neuronal death since both LC3-II expression and SQSTM1 degradation were increased at 3 and 6 h. In order to confirm an enhanced autophagic flux, ibotenate was first applied in the presence of lysosomal enzymes inhibitors (Fig. 6c). A combination of E64 and pepstatin A1 (PepA) (10 μg/ml) induced an accumulation of both LC3-II (of 123%) and SQSTM1 (of 13%) reflecting the failure in the autophagy degradation step. When ibotenate was applied 4 h after E64/PepA, LC3-II and SQSTM1 accumulations were even greater (174 and 21% respectively), demonstrating that ibotenate treatment triggered the new formation of autophagosomes and thus confirming that ibotenate treatment increased the autophagosome biogenesis. Second, autophagic flux was monitored using the tandem mRFP-GFP-LC3 plasmid that allows to discriminate between LC3 expressed in neutral compartments (GFP + RFP+; early autophagosomes: i.e. autophagosomes before fusion with lysosomes) and in acidic vesicles (GFP-RFP+; i.e., late autophagosomes: autophagosomes after fusion with lysosomes (autolysosomes)) thanks to the pH sensitivity differences exhibited by the two fluorescent proteins (Fig. 6e). A quantification of the different LC3-positive dots per neuron per µm^2^ clearly demonstrated that both autophagosomes formation (~5 fold increase of GFP + RFP + dots) and their fusion with the lysosomes (~13 fold increase of GFP−RFP + dots) were enhanced by ibotenate treatment. Taken together, these results on cortical neuronal cultures allow to conclude that an excitotoxic dose of ibotenate was efficient to induce a boost of neuronal autophagic flux.

### Pharmacological and genetic inhibition of autophagy is protective against ibotenate-induced excitotoxicity in primary cortical neuronal cultures

**Fig. 7:**
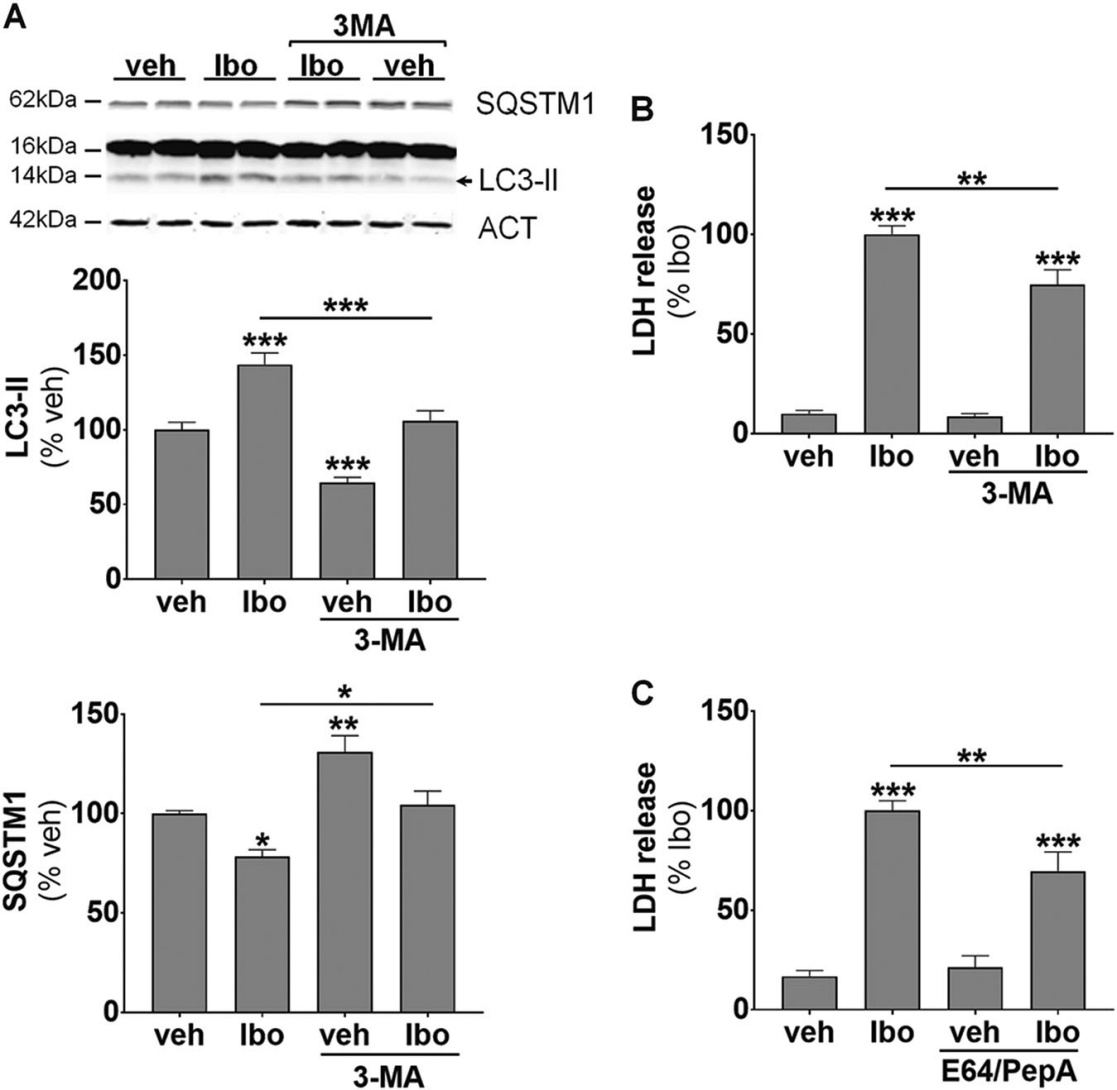
Pharmacological inhibition of autophagy is protective against ibotenate-induced excitotoxicity in primary cortical neuronal cultures. **a** Pretreatment with 3-methyadenine (3-MA) 1 h before ibotenate (Ibo) addition for 6 h prevents LC3-II increase (veh: 100 ± 5%; Ibo: 143.7 ± 7.8%; veh + 3-MA: 64.6 ± 3.4%; Ibo + 3-MA: 106.1 ± 6.7%) and SQSTM1 decrease (veh: 100 ± 1.4%; Ibo: 78.4 ± 3.4%; veh + 3-MA: 131 ± 8.2%; Ibo + 3-MA: 104.3 ± 7.1%) induced by Ibo as shown by representative immunoblots and the corresponding quantifications. **b** 3-MA decreases neuronal death as shown by reduced lactate deshydrogenase (LDH) release in the culture medium of cultured neurons (veh: 10 ± 1.7%; Ibo: 100 ± 4.3%; veh + 3-MA: 8.6 ± 1.4%; Ibo + 3-MA: 74.7 ± 7.5%). **c** E64/PepA pretreatment reduces neuronal death as indicated by a decrease in LDH release measured 6 h after Ibo addition (veh DMSO: 16.8 ± 2.9; Ibo DMSO: 100 ± 5%; veh E64/PepA: 21.3 ± 5.8%; Ibo E64/PepA: 69.6 ± 9.7%). Values are mean ± SEM, *p < 0.05; **p < 0.01; ***p < 0.001

We then assessed the functional role of the ibotenate-enhanced autophagy in primary cortical neuronal cultures. We first used 3-MA. Pre-treatment with 3-MA (10 mM) prevented the increases in both LC3-II expression and SQSTM1 degradation at 6 h after ibotenate treatment (Fig. 7a). 3-MA displayed a significant neuroprotective effect as shown by a decrease of 26% in LDH release (Fig. 7b). Interestingly, blocking lysosomal degradation with E64/PepA also reduced neuronal death (Fig. 7c) suggesting a role of autophagy degradation step in ibotenate-induced neurotoxicity.

**Fig. 8:**
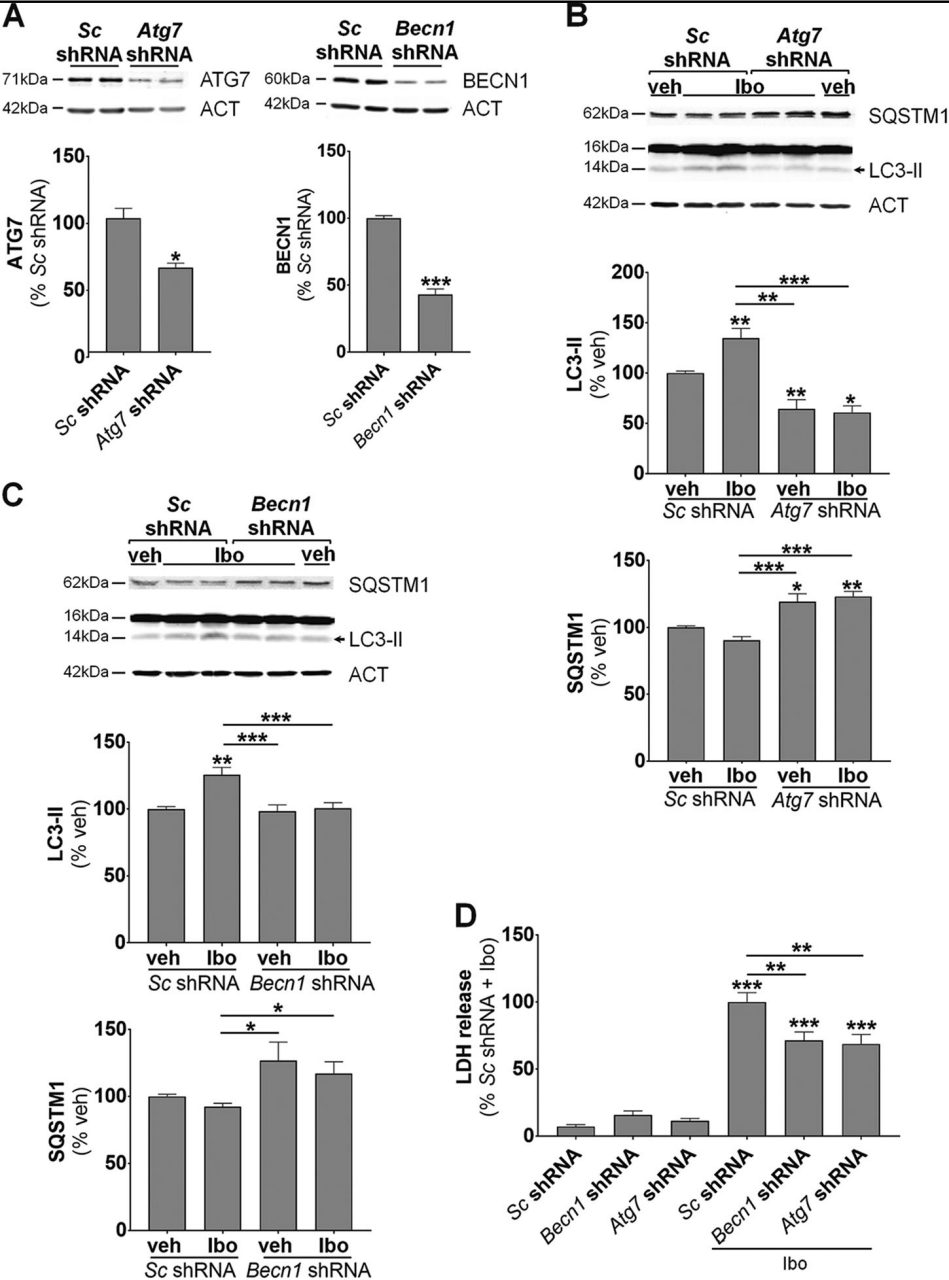
Genetic inhibition of autophagy is protective against ibotenate-induced excitotoxicity in primary cortical neuronal cultures. **a** Lentiviral transduction of shRNA directed against BECLIN1 (BECN1) (*sc* shRNA: 100 ± 1.9%; *Becn1* shRNA: 43 ± 12.3%) and ATG7 (*sc* shRNA: 100 ± 7.4%; *Atg7* shRNA: 63 ± 10.9%) proteins efficiently reduced the expression of both proteins. **b**, **c** Downregulation of both (**b**) BECN1 and (**c**) ATG7 prevents LC3-II increase ((**b**) veh *sc* shRNA: 100 ± 2.1%; Ibo *sc* shRNA: 135 ± 9.4%; veh *Atg7* shRNA: 64.4 ± 9.1%; Ibo *Atg7* shRNA: 60.8 ± 6.6%; (**c**) veh *sc* shRNA: 100 ± 1.8%; Ibo *sc* shRNA: 125.6 ± 5.5%; veh *Becn1* shRNA: 98.4 ± 4.6%; Ibo *Becn1* shRNA: 100.6 ± 4.1%) and SQSTM1 decrease ((**b**) veh *sc* shRNA: 100 ± 1.4 %; Ibo *sc* shRNA: 93 ± 3.4%; veh *Atg7* shRNA: 109.5 ± 4%; Ibo *Atg7* shRNA: 117.2 ± 3.6%; (**c**) veh *sc* shRNA: 100 ± 1.4%; Ibo *sc* shRNA: 91.3 ± 3.2%; veh *Becn1* shRNA: 132.4 ± 19.3%; Ibo *Becn1* shRNA: 119.4 ± 10.7%) induced by Ibo (6 h) as shown by representative immunoblots and the corresponding quantifications. **d** Downregulation of both ATG7 and BECN1 has a neuroprotective effect as demonstrated by reduced lactate deshydrogenase (LDH) release in the culture medium of cultured neurons (veh *sc* shRNA: 7.2 ± 1.4 %; veh *Atg7* shRNA: 11.3 ± 1.9%; veh *Becn1* shRNA: 15.9 ± 3%; Ibo *sc* shRNA: 100 ± 7%; Ibo *Atg7* shRNA: 71.4 ± 6.3%; Ibo *Becn1* shRNA: 68.8 ± 7%). Values are mean ± SEM, *p < 0.05; **p < 0.01; ***p < 0.001

Then, in order to inhibit more specifically autophagy, downregulation of the expression of two important autophagy proteins (ATG7 and BECLIN1 (BECN1)) was performed using lentiviral vectors transducing short hairpin RNAs (shRNAs) (Fig. 8a). Transduction of cultured primary cortical neurons with *Atg7* and *Becn1* shRNAs resulted in an efficient inhibition of autophagy as demonstrated by a decrease in both LC3-II expression and SQSTM1 degradation (Figs. 8b, c). The death-promoting role of enhanced autophagy in ibotenate-induced neuronal death was confirmed by a reduction of ~30% of LDH release when ATG7 and BECN1 were downregulated (Fig. 8d).

In conclusion, these in vitro data confirmed that an excitotoxic dose of ibotenate could induce autophagy-mediated neuronal death and that targeting autophagy inhibition could lead to neuroprotection.

## Discussion

In the present study, we investigated for the first time the role of autophagy in a preclinical model of premature brain damage related to cPVL^42^. The effect of ibotenate injection on rodent brain development is highly dependent of the age. If injected around P5, when neuronal migration is completed, the model mimics some of the preterm brain injury features^42^. Human preterm WM injury is often observed with GM developmental alteration and/or damage (neuronal-axonal disease)^2,3,16,17,44^. Persistent cerebral volume reduction and ventriculomegaly are also observed in premature infants compared to full-term^45,46^. The injection of ibotenate in the subcortical WM at the level of the right cingulum caused a severe lesion in rat pups and led to reduced subcortical WM thickness, significant loss of tissue and important lateral ventricle enlargement 16 days after injury. It has been shown that excitotoxicity-induced inflammation through microglial release of cytokines and free radicals played a central deleterious role^47,48^ and that apoptotic pathways were implicated in similar models of preterm brain injury^49,50^. However, autophagy-mediated neuronal death has never been investigated in this context.

Macroautophagy is an important physiological mechanism of degradation present at a basal level complementary to the proteasome system. A “controlled” upregulation of autophagy has been considered for a long time as a survival response, for instance, acting as an alternative source of energy during starvation or as a quality control step eliminating toxic metabolites, defective organelles or intracellular pathogens^23,51,52^. However, this dogma has been challenged about 30 years ago by the description of dying cells without typical morphological hallmarks of apoptosis or necrosis and containing numerous autolysosomes^27^. This new morphological type of cell death was coined “autophagic cell death” and the missuse of the term led to a strong debate on a possible pro-death role of autophagy in some stress conditions^10,34,35^. In fact, “autophagic cell death” is a distinct mechanism of cell death independent of apoptotic and necrotic machinery that is observed in some specific circumstances^27,38,53^. However, there is now evidences and it is well accepted, that autophagy can be more frequently involved in cell death as a trigger leading to apoptotic or necrotic cell death (autophagy-mediated cell death)^20,21,29,34,38,54–57^. Excitotoxicity and cerebral HI are some of the conditions where autophagy is enhanced^58^. Although some controversies exist^32,33,59^, most of the studies using pharmacological inhibitors such as 3-MA^30,37^ and especially those using specific and genetic inhibition of *atg*^20,29,31^ revealed a deleterious role of autophagy. They also supported the concept of a strong interconnection between autophagic and apoptotic mechanisms in perinatal cerebral HI^19,20,32,60^. The present study is the first to demonstrate a death-promoting role of autophagy in a preterm model of excitotoxic brain lesion.

Our results strongly suggest that autophagy is enhanced in neurons after ibotenate injection in the brain of rat pups, as also demonstrated in primary cortical neurons cultures. Autophagosome formation is increased from 6 h as shown by a higher level of LC3-II and more LC3-positive dots. This increase was not due to impaired autophagosome degradation that would have occurred if lysosomal function was defective. In fact, p62/SQSTM1 is also degraded and autolysosomes are increased in neurons as indicated by larger and more numerous LAMP1- and CTSB-positive dots. This result is consistent with EM observations showing the presence of both autophagosome-like multimembrane vesicles and autolysosome-like dense structures in dying neurons of ibotenate-injected rat pups. We provide here, especially in primary neuronal cultures, compiling evidences demonstrating that the autophagy flux is increased by ibotenate treatment. Both Western blots against LC3 and p62/SQSTM1 with or without E64/pepstatinA co-treatments and the use of the GFP-RFP-LC3 construct lead to the same conclusion, ibotenate treatment is increasing autophagosome formation and degradation. Moreover, the GFP-RFP-LC3 construct we used is less sensitive to acidification comparing to other constructs, such as the mKate2-pHluorin-hLC3^61^, suggesting that the number of autolysosomes could be theoretically underestimated. Our in vitro data on primary cortical neuronal cultures also strongly argued for the induction of an autophagy-mediated neuronal death by ibotenate. Inhibition of autophagy, not only pharmacologically with 3-MA or E64/PepA, but also genetically by downregulating two important ATG proteins, ATG7 or BECN1, was neuroprotective as previously shown when excitotoxicity was induced in hypoxic conditions^29^.

Even if caution is necessary concerning the limited specificity of 3-MA as an autophagy inhibitor^22^, we here clearly showed that the dose used in vivo efficiently prevented autophagy (decrease in LC3-II level and SQSTM1/p62 degradation). Furthermore, since it is known that permanent impaired autophagy leads to neurodegeneration (as it would be the case with long term genetic inhibition of autophagy)^43^, the use of 3-MA was appropriate to study long term effect on brain lesion in vivo (16 days after the insult). When ibotenate-enhanced autophagy was prevented by 3-MA treatment, both CASP3 activation and calpain-dependent cleavage of SPTAN (used as an indicator of necrotic cell death characterized by a calcium increase) were significantly decreased. Moreover, ultrastructural observations showed condensed chromatin in nuclei of dying and highly autophagic neurons. Mixed features of apoptosis and autophagy were also observed in rodent models of perinatal cerebral HI^19,20,30,31,60^ and we previously demonstrated that autophagy could contribute to apoptosis using widely recognized apoptotic stimuli in primary cortical neuron cultures^62^. The beneficial effect of 3-MA on neuronal cell death, including apoptosis, resulted here in a strong significant neuroprotective effect at long term on both GM and WM, suggesting a crucial role of autophagy in mediating (apoptotic and necrotic) cell death. Studies on perinatal cerebral HI models have also shown that autophagy inhibition could be neuroprotective and reduce apoptosis^20,30,31^ suggesting that the transient inhibition of autophagy could be a promising strategy to protect the immature brain against excitotoxic insults.

It has been shown that females are more resistant to perinatal cerebral injury than males, especially in the context of cerebral HI and after moderate lesions, in rodents as well as in humans^63–66^. The reason of this gender difference is still unclear, but sex-dependent cell death pathways have been recognized after perinatal cerebral HI, especially more active caspase-dependent pathways in females^66–68^. In the present study, we also found a stronger CASP3 activation and a more important variability in females in almost all the different parameters investigated. However, mean ibotenate-induced brain lesion volume was similar in both genders. Moreover, the protective effect of 3-MA against both ibotenate-induced autophagy and cerebral lesions was as efficient in males as in females.

In conclusion, we showed for the first time that enhanced autophagy could mediate cell death in a premature model of excitotoxic brain damage. Autophagy inhibition in this severe model is very promising since the protective effect obtained is similar or even better to other previously described neuroprotectants such as caspases inhibitors^69^, erythropoietin^70,71^, BDNF^72,73^, melatonin^74^ or magnesium sulfate^75,76^. Interestingly, we recently demonstrated that autophagy is enhanced in dying neurons in the ventrolateral nucleus of the thalamus and the lentiform nucleus of term newborns with severe HIE^31,60^. Apoptotic markers were also expressed in dying highly autophagic neurons, arguing for a possible association between autophagy and apoptosis also in humans. Moreover an increased presence of autophagosomes (LC3-positive dots) was recently demonstrated in WM injury of extremely preterm infants^77^. These 3 different studies using human newborn brain sections^31,60,77^ and the important neuroprotection obtained with 3-MA in the present study in a severe model of cPVL suggest that enhanced neuronal autophagy could be a promising target. The development of strategies transiently inhibiting autophagy could pave the way for new therapies against neonatal severe excitotoxic injuries such as HIE and cPVL.

## Material and methods

### Primary cortical neuronal cultures

Primary neuronal cultures were prepared according to the Swiss laws for the protection of animals from pieces of cortices of 2-day-old Sprague-Dawley rat pups (Janvier Labs, Mayenne, France). The procedures were approved by the Vaud Cantonal Veterinary Office. After dissection, dissociation and trituration, neurons were plated in neurobasal medium (Gibco, NY, USA; 21103–049) supplemented with 2% B27 (Gibco; 17504044), 0.5 mM L-glutamine (Sigma, MO, USA; 49419) and 100 μg/ml penicillin-streptomycin (Gibco; 15140122) and maintained at 37 °C with a 5% CO_2_-containing atmosphere as described previously^78^. Western blot analyses were done on neurons plated at a density of ~7 × 10^5^ cells/dish (35-mm poly-D-lysine pre-coated dishes (BD Biosciences, NJ, USA; 356467) and at a density of ~3 × 10^5^ cells on 12-mm glass coverslips coated with 0.01% poly-L-lysine (Sigma; P4832) for immunocytochemistry and imaging. For all the Western blots or imaging results, at least three independent experiments, each involving two or three culture dishes or coverslips, were performed.

#### Pharmacological treatments

Primary cortical neuronal cultures were pre-treated for 1 h with 10 mM 3-methyladenine (3-MA) (Sigma; M9281), 5 mM EGTA (Sigma; E0396) or 40 μM MK801 (Sigma; M107). For inhibition of lysosomal degradation, a cocktail of 10 μg/ml pepstatin A1 (PepA) (Sigma; P5318) and 10 μg/ml E64 (Sigma; E3132) was applied for 4 h prior to 50 μM ibotenic acid (Enzo Life Sciences, NY, USA; BML-EA120–0001) addition.

#### Quantification of cell death with lactate dehydrogenase release

Cell death was assayed by measurement of LDH released in the medium using the Cytotox 96 nonradioactive cytotoxicity assay kit (Promega, WI, USA; G1780) as previously described^78^. LDH measurements were normalized with respect to the values of ibotenate-treated neurons 6 h after the ibotenate addition.

#### Knockdown of ATG using lentiviral vectors

Downregulation of *Atg* genes were performed with pLKO lentiviral vectors (Open Biosystems/Dharmacon, CO, USA) expressing rat-specific shRNA sequences from TRC (the RNAi consortium) library as described previously^62^. A combination of TRCN0000092163 and TRCN0000092166 for *Atg7* (GenBankTM NM_001012097), TRCN0000033552 for *Becn1* (GenBankTM NM_053739.2) and a pLKO vector containing scrambled shRNA (Open Biosystems/Dharmacon) as control vector were used. Primary cortical neuron cultures were infected at DIV7 with 50 ng of the viral capsid protein p24/ml culture medium for each vector.

#### mRFP-GFP-LC3 plasmid transfection and quantification

Neurons on coverslips were transfected with the tandem mRFP-GFP-LC3–expressing plasmid ptfLC3 (Addgene, MA, USA; 21074)^79^ using Lipofectamine 2000 (Invitrogen, CA, USA; 11668-019) as described previously^29^. Coverslips were fixed with 4% paraformaldehyde for 15 min. Confocal images were acquired using a Zeiss LSM 710 Meta confocal laser scanning microscope. Total LC3-positive dots (GFP^+^ RFP^+^ and GFP^−^ RFP^+^ dots), early autophagosomes (GFP^+^ RFP^+^ dots) and mature or late autophagosomes (GFP^−^ RFP^+^ dots) were analyzed using ImageJ software and expressed as a number of positive dots per neuron per µm^2^.

### Rat model of preterm excitotoxic brain injury

All experiments were performed in accordance with the Swiss laws for the protection of animals and were approved by the Vaud Cantonal Veterinary Office. Ten µg of ibotenate (diluted 5 µg/µl in acetic acid 0.02%) were stereotaxically injected under isoflurane anesthesia (2.5%) in the subcortical WM at the level of the right cingulum (1 mm posterior and 1 mm right from Bregma and 1.5 mm depth from the skull surface) of 5-day-old male and female Sprague Dawley rats (Janvier Labs) (model adapted from Marret and colleagues (1995)^42^). The control animals received an injection of the same volume of vehicle (acetic acid 0.02%). The pharmacological autophagy inhibitor 3-MA (2 µl of 30 mg/ml in saline) was stereotaxically injected in the right lateral ventricle (0.5 mm posterior and 1 mm right from Bregma and 2.5 mm depth from the skull surface) just before ibotenate injection. Control animals received an injection of the same volume of saline as vehicle. After recovering from anesthesia, rat pups returned to the dam until sacrifice.

### Electron microscopy

Electron microscopy (EM) was done on rat brains fixed following intracardiac perfusion with 2.5% glutaraldehyde and 2% paraformaldehyde in cacodylate buffer (0.1 M, pH7.4) as previously described^80^.

### Immunoblotting

Immunoblots were performed on extracts from primary neuronal cultures or from cortex collected in lysis buffer (20 mM HEPES, pH 7.4, 10 mM NaCl, 3 mM MgCl_2_, 2.5 mM EGTA, 0.1 mM dithiothreitol, 50 mM NaF, 1 mM Na_3_VO_4_, 1% Triton X-100 and a protease inhibitor cocktail (Sigma;11873580001)^29^. Protein concentration was determined using a Bradford assay. Proteins (20–40 µg) were separated on 10, 12 or 15% polyacrylamide gels and analyzed by immunoblotting. Antibodies were diluted in the blocking solution containing 0.1% casein (Sigma; C8654). Primary antibodies used were: anti-ATG7 (sc-33211) rabbit polyclonal and anti-BECN1 (sc-11427) rabbit polyclonal from Santa Cruz Biotechnology (Santa Cruz, CA, USA); anti-LC3 (ab48394) rabbit polyclonal from Abcam (MA, USA); anti-cleaved CASP3/caspase-3 (9661) rabbit polyclonal from Cell Signalling Technology (MA; USA); anti-SQSTM1 (P0067) rabbit polyclonal from Sigma; anti-FODRIN/SPECTRIN (FG6090) mouse monoclonal from Enzo Life Sciences and anti-ACTA/α- ACTIN (MAB1501) mouse monoclonal from Millipore/Merck (MA, USA). Secondary antibodies were polyclonal goat anti-mouse or anti-rabbit IgG from LiCOR (IRDye 680 or IRDye 800). Protein bands were visualized with the Odyssey Infrared Imaging System (LICOR, NE, USA). Odyssey v1.2 software (LICOR) was used for analysis. Values were normalized with respect to ACTIN.

### Immunohistochemistry

Immunostainings were performed on 20μm cryostat brain sections from rat pups perfused transcardially with 4% paraformaldehyde in 0.1 mol/L PBS (pH 7.4)^29^. PBS with 15% donkey serum and 0.3% Triton X-100 were used for blocking and permeabilization for 30–45 min. Primary antibodies diluted in PBS with 1.5% donkey serum and 0.1% Triton X-100 overnight at 4 °C were: anti-cleaved CASP3/caspase-3 (9661) rabbit polyclonal from Cell Signalling Technology; anti-LC3 (ab48394) rabbit polyclonal from Abcam; anti-CTSB/cathepsin B (06–480), anti-MAP2 (AB5622) rabbit polyclonal antibodies, anti-RBFOX3/NeuN (MAB377) and anti-LAMP1 (428017) mouse monoclonal antibodies from Merk/Millipore. Secondary antibodies were diluted in PBS for 2 h at room temperature.

For immunofluorescence labeling, Alexa Fluor donkey-anti-rabbit or mouse secondary antibodies (Invitrogen; A21202, A21203, A21206, A21207) were used. A LSM 710 Meta confocal microscope (Carl Zeiss) were used for confocal laser microscopy. Images were processed with LSM 510 software and mounted using Adobe Photoshop.

### Quantification of autophagic and lysosomal labeling

Confocal images of fluorescent immunostaining against LC3, CTSB and LAMP1 were acquired using the LSM 710 Meta confocal laser scanning microscope (Carl Zeiss) and images were then processed with Adobe Photoshop CC 2015. Positive dots were quantified using ImageJ software and expressed as a number of positive dots per neuron per μm^2^ and, for the lysosomal markers CTSB and LAMP1, as a mean dot area per neuron per μm^2^.

### Cerebral regions volume and WM thickness measurements

Sixteen days after the injury (at P21) brains were perfused, frozen and entirely cut into series of 20 μm coronal sections spaced at 500μm disposed in series. On a cresyl violet-stained series, the ipsilateral volume of the total surviving tissue, the cortex and the lateral ventricle were measured using the Zen Blue software (Zeiss). The volumes were then expressed as a percentage of the total brain volume.

WM thickness were measured on 3 consecutive cresyl violet-stained sections starting from the first on which the genu of the corpus callosum appeared (approx. 0.6–0.8 mm anterior to the Bregma according to the “atlas of the rat brain in stereotaxic coordinates at P21” of Khazipov et al., http://www.ialdevelopmentalneurobiology.com/images/atlases/Atlas-p21.pdf^81^). The thickness of the ipsilateral corpus callosum (on the midline) and the subcortical WM at the level of the cingulum (1.4 mm apart from the midline) and at the beginning of the external capsule (2 mm apart from the midline) were measured parallel to the midline with the Zen Blue software (Zeiss). Values are expressed as a mean of the three measures.

### Statistics

Values were expressed as mean values ± standard error of the mean (SEM). Data were analyzed statistically using GraphPad PRISM (version 7.03) software. The normality of the distribution was first tested using Shapiro–Wilk tests. Parametric data were analyzed using a Welch’s ANOVA test (one-way ANOVA with unequal variances) followed by a post-hoc Tukey-Kramer test. Non-parametric data were analyzed using a Kruskal–Wallis test (non-parametric analog of the one-way ANOVA) followed by a post-hoc Steel-Dwass. *P* < 0.05 was chosen as threshold for statistical significance.
